# COVID-19 cancer conundrum—evidence driving decisions or the lack of it?

**DOI:** 10.1186/s12916-020-01649-z

**Published:** 2020-06-09

**Authors:** Nalinie Joharatnam-Hogan, Khurum Khan

**Affiliations:** 1 Gastrointestinal Oncology Service & Cancer of Unknown Primary Service, University College London Hospital NHS Foundation Trust, London, UK; 2 UCL Cancer Institute, London, UK; 3 North Middlesex University Hospital, London, UK; 4 MRC CTU UCL, London, UK

**Keywords:** COVID-19, Oncology research, Chemotherapy, Public health, Cancer care, Cancer screening

## Background

The novel coronavirus SARS-CoV-2, the causative pathogen of the COVID-19 pandemic, is characterised pathologically by inflammation, a feature considered to be a hallmark of another uncontrolled pandemic, “cancer”. Whilst these two diseases are separate entities with disparate aetiologies, they share some overlapping features—including multi-organ involvement, widespread inflammation, and a paucity of definitive treatment options. The pandemics are also comparable in their scale. The World Health Organisation (WHO) reported 18.1 million new global cancer cases and 9.6 million cancer-related deaths in 2018, with COVID-19 having 4.7 million cases and 316,289 fatalities as of 19 May 2020 [1]. There has been a decades-long drive to improve cancer outcomes, utilising the expertise of cancer researchers, epidemiologists, translational and clinical scientists, immunologists, statistical modellers, and health economists. The urgent, round-the-clock race to develop a vaccine or feasible treatment for COVID-19 is involving a similar multi-discipline effort.

## Examining the evidence

There is a notion that cancer patients, due to their higher average age, prevalence of co-morbidities, and immunosuppression, may be at higher risk of increased morbidity and mortality associated with COVID-19. There is particular concern for those receiving chemotherapy. Current evidence supporting this concern remains inconclusive and is an active focus of ongoing research. Due to the high prevalence of cancer patients in the UK, many of whom are receiving chemotherapy, we recognise an urgency in defining any excess risk of COVID-19 and cancer.

A prospective nationwide analysis of 1590 patients with COVID-19 in China, of whom 1% had a history of cancer, has shown an increased likelihood of having a severe event (a composite of invasive ventilation or death) in those patients with cancer (39% versus 8% in patients without cancer, *p* = 0.0003) [2]. These data must be interpreted with caution due to the small sample size of cancer patients (*n* = 18) and significant heterogeneity between these tumour types. A subsequent study from Wuhan comparing 105 patients with cancer to 536 age-matched patients without cancer demonstrated a higher frequency of severe events related to COVID-19 particularly in patients with haematological malignancies, lung cancer, or metastatic disease [3]. Our group formed a North London collaboration of five hospitals and found no significant differences in mortality of two consecutive cohorts comprising of COVID-19 positive cancer and non-cancer patients [4]. It is possible there may be a confounding factor of chemotherapy-associated immunosuppression having a protective effect against some of the inflammatory processes of COVID-19 by virtue of dampening the cytokine storm. All of these studies had significant limitations due to the retrospective nature of the data, heterogeneity amongst the patient population, and lack of pre-determined study parameters, which may offer logical explanation for the conflicting results.

## Cancer care compromised

The surge of COVID-19 cases threatened to overwhelm acute health services in almost every country in the world. To create the necessary critical care capacity, countries such as the UK, Italy, and the USA were required to re-structure non-acute services. Patients with cancer worldwide have faced disruption of their care, including delaying of urgent procedures, deferral of surgery, discontinuation of systemic therapy, and modification of standard radiotherapy procedures [5]. Cancer screening programmes have been paused and cancer diagnoses heavily impacted. In the UK, a 25% reduction in urgent cancer referrals has been recorded, estimated to equate to 2300 undiagnosed cases every week [6]. Mirroring the pandemic caseload, there has been a surge of novel clinical trials assessing both vaccine and treatment candidates for COVID-19. However, there has been a precipitous reduction in cancer trial recruitment and development.

The rationale for modifying cancer services is underpinned by the balance of clinical need and significant possible risks to patients from the COVID-19 infection, as well as the need to optimise already stretched resources and capacity [7]; however, concerns remain about the likely impact on individual cancer care and cancer mortality. Warnings have been made about a second wave of COVID-19 infections [8], which theoretically could coincide with a wave of delayed presentations of advanced cancer or early progression of disease.

## What the future holds

Re-establishing gold-standard cancer care and research in the post-COVID-19 era is likely to be challenging. Research is currently being undertaken to determine what changes to care delivery and clinical research, implemented in response to COVID-19, could be retained, and how delivery of care can be amended [9]. Data is required on the likely impact COVID-19 will have on morbidity and mortality. It has been estimated that there will be a 20% increase in deaths occurring over a year in England in patients with new cancer diagnoses as result of the COVID-19 pandemic, equivalent to an additional 6270 deaths [10]. Prospectively collected precise data on the alterations made to cancer services and true mortality of these patients is required to better understand the potentially severe unintended consequences of the response to the COVID-19 pandemic, to inform the appropriate management and prioritisation of healthcare services.

## Conclusion

We are entering a recovery phase of the COVID-19 pandemic; however, it is inevitable that cancer services will not resume in the manner they did in the pre-COVID era. Healthcare services need to adapt to the presence of both global burdens, cancer and COVID, to minimise complications from either. The generation of timely evidence on the impact of COVID-19 on cancer care and patient outcomes is required to guide future cancer care delivery and cancer research. We need to ensure ongoing sustainability and safety of future management pathways and enable a coordinated response of all overlapping specialities in the fight against this and any future pandemics.

## Data Availability

Not applicable

## Abbreviations

WHO: World Health Organisation
